# Perceptual load and early selection: an effect of attentional engagement?

**DOI:** 10.3389/fpsyg.2013.00498

**Published:** 2013-08-02

**Authors:** Karina J. Linnell, Serge Caparos

**Affiliations:** ^1^Department of Psychology, Goldsmiths, University of LondonLondon, UK; ^2^Department of Psychology, Université du Québec à Trois-RivièresQuébec, QC, Canada

The selection of task-relevant information from amongst task-irrelevant or distracting information is key to successful performance, and much debate has focused on the processing stage(s) at which this selection takes place. Early-selection theory claimed that the selection of task-relevant information occurs at an early perceptual level of processing, so that only targets are perceptually encoded (Cherry, 1953; Broadbent, 1958). In contrast, late-selection theory claimed that both targets and distractors are perceptually encoded and that target selection occurs at a late post-perceptual level of processing (DeutSch and DeutSch, 1963). Lavie (1995) attempted to reconcile these theories by suggesting that early and late selection occur, respectively, when the perceptual load associated with the selection of the target is high and low.

Thus, according to load theory, when the perceptual load associated with target selection is high, perceptual resources are completely exhausted with perceptual processing of the target and unavailable for perceptual processing of distractors; conversely, when the perceptual load associated with target selection is low, perceptual resources are not completely occupied with perceptual processing of the target and automatically spill over to allow perceptual processing of distractors. In sum, load theory suggests that early selection is possible only when perceptual capacity is exhausted. When capacity is not exhausted, post-perceptual mechanisms must be invoked to inhibit irrelevant information that received perceptual processing (Macdonald and Lavie, 2008).

The majority of studies providing evidence in support of load theory have used the flanker paradigm which presents distractors and targets at fixed separations (Eriksen and Eriksen, 1974) and have shown that higher perceptual load generates lower distractor interference. These studies interpret lower distractor interference under high perceptual load as an expression of spatial attention that is more narrowly focused on the target. In order to test this interpretation directly, we presented distractors at varying separations from targets in a variant of the flanker paradigm (Eriksen and St. James, 1986) and measured distractor interference as a function of separation to index the spatial profile of attention. In addition, and following Lavie (1995), we manipulated perceptual load using not just the more standard stimulus-based manipulations (involving varying the number of filler items surrounding the to-be-identified target item) but also task-based manipulations (involving varying the spatial-resolution difficulty of a secondary perceptual task performed after target identification). Note that stimulus-based manipulations are possibly confounded with “dilution” of distractors (Tsal and Benoni, 2010). For both types of perceptual load manipulations, however, we showed that the spatial profile of attention was more focused when perceptual load was high and less focused when it was low (Caparos and Linnell, 2009, 2010; Linnell and Caparos, 2011), consistent with the central tenet of load theory that perceptual load affects early perceptual-level selection.

Critically, however, high perceptual load only focused spatial attention when working memory load was low (i.e., when one, as opposed to six, digits were held in memory) and, thus, when cognitive resources were available (Linnell and Caparos, 2011). This finding is not consistent with the claim of load theory that high perceptual load focuses spatial attention automatically (and with the finding of Lavie et al., 2004, that perceptual and working memory load exert independent effects on distractor interference; see discussion of this finding in Caparos and Linnell, 2010, and in Linnell and Caparos, 2011). The fact that perceptual load only focuses spatial attention when cognitive resources are available raises the possibility that perceptual load is important in early selection not because it exhausts perceptual resources but rather because it engages cognitive resources sufficiently on the task in hand to focus spatial attention.

The requirement for cognitive resources may not be great since even groups that demonstrate impairments in cognitive control such as the elderly and high-trait-anxious can show levels of selection indistinguishable from their younger and low-trait-anxious counterparts under high perceptual load (Maylor and Lavie, 1998; Bishop, 2009). We argue that increasing perceptual load increases task difficulty in a straightforward fashion that only impacts perceptual difficulty; this does not challenge cognitive resources but simply engages them in the focusing of spatial attention (see also the suggestion of Eysenck and Derakshan, 2011, that “when the task is demanding and there are clear task goals, high-anxious individuals have a high level of motivation [and make] extensive use of attentional control strategies”). A high-perceptual-load task is according to this conception a task that perceptually draws one in; it encourages the investment of cognitive resources - that might otherwise have been spent on mind wandering or other distractions - on the focusing of spatial attention. Compatible with this, perceptual load has been reported to decrease self-reported mind-wandering and to improve selection in proportion to improvements in mind-wandering (Forster and Lavie, 2009).

In essence, we are arguing that perceptual load—whether it is manipulated using stimulus or task complexity—is a perceptual-difficulty manipulation that motivates cognitive engagement with a task. This is compatible with the common finding that perceptual load exerts considerably larger or more consistent effects on selection when it is blocked rather than varied from trial to trial (e.g., Theeuwes et al., 2004) but can also explain the smaller residual effects of load on a trial-by-trial basis. Although increasing perceptual load will increase perceptual difficulty, what is difficult and engaging to one individual or group of participants may not be difficult and engaging to another. Computer gamers and ASD participants have both been argued to possess more perceptual resources than controls and their greater distractibility at lower perceptual loads (Green and Bavelier, 2003; Remington et al., 2009) may be explained by these loads not being sufficiently perceptually difficult to induce cognitive engagement. This is a different account from that offered by load theory which does not invoke any differences in cognitive engagement; according to load theory, the key difference between groups that differ in their perceptual capacity is whether or not fixed-capacity perceptual resources are exhausted (Maylor and Lavie, 1998).

As soon as one explains the effect of perceptual load on early perceptual-level selection as an effect of attentional engagement one should allow that conditions of high perceptual load may not be the only ones supporting early selection; even when the perceptual difficulty of the task is low, early selection may be possible and distractors may not be perceptually processed. This is exactly what was found in a task of local selection where we showed that increasing task interest by increasing social relevance, without changing perceptual load, focused spatial attention and thus improved early perceptual-level selection (Linnell et al., 2013). In addition, groups that differ in their emotional responses and default attentional state also differ in their propensity for early perceptual-level selection: in a task of local selection with no emotional content, high-trait-anxious participants displayed more focused spatial attention than low-trait-anxious ones (Caparos and Linnell, 2012). High-trait-anxious individuals are likely to make more effort in order to avoid failure at a task (Staal, 2004; Sarter et al., 2006), resulting in cognitive resources being more engaged to focus spatial attention.

Differences in default attentional state may also underpin the far greater facility for early selection in remote peoples compared to urbanized peoples. The Himba are a remote people living in the savannah of northern Namibia; in a range of tasks of local selection, they consistently displayed more focused spatial attention than British participants living in London and than Himba who had adopted an urbanized way of life (Linnell et al., 2013). This was not a result of the Himba having fewer perceptual resources because they showed the same sensitivity to increasing perceptual load as British participants and showed focused spatial attention even at the lowest perceptual load (De Fockert et al., 2011). Rather, we argue that remote peoples have a default attentional state that favors full cognitive engagement with the task in hand, whereas urbanized peoples—perhaps because they are more stressed—have a default attentional state that favors the division of cognitive resources between monitoring the wider environment for dangers or opportunities and performing the task in hand (Linnell et al., 2013).

Our explanation of these default differences between urbanized and non-urbanized groups invokes a variant of the Yerkes-Dodson law (Yerkes and Dodson, 1908) that links task performance to attentional state by an inverted U-shaped function. According to this law, task performance—here attentional selection—peaks at an intermediate attentional state. This intermediate state may not be the default attentional state, at least in the urbanized peoples who have participated in most studies of attention. The default attentional state in urbanized peoples may favor late over early selection; processing contextual information at least to perceptual levels may be advisable in complex and dynamic urban environments—where distractors can suddenly become targets—and therefore worth the cost when distractors have to be ignored at post-perceptual levels in the service of task goals. Nevertheless, we are just starting to understand how altering the motivational significance of the task in hand may shift this balance (e.g., Padmala and Pessoa, 2011).

In sum, we suggest that altering the perceptual load associated with target processing—whether by stimulus-based or task-based manipulations—is just one of a number of ways of affecting the engagement of cognitive resources with the task in hand and that it is increasing engagement with a target stimulus that is key to achieving its early perceptual-level selection and not the exhausting of perceptual capacity. Attentional engagement and early perceptual selection (i) may be the default in remote peoples even when perceptual load is low and there are no special motivating factors and (ii) may be demonstrable in urbanized peoples when they are confronted not just with high perceptual load but also with tasks of social or emotional significance. It is time not just to bring the study of attention and motivation together, but to recognize that motivation is key to a core construct of attention research, namely perceptual load.
